# Prevalence and unmet need for diabetes care across the care continuum in a national sample of South African adults: Evidence from the SANHANES-1, 2011-2012

**DOI:** 10.1371/journal.pone.0184264

**Published:** 2017-10-02

**Authors:** Andrew Stokes, Kaitlyn M. Berry, Zandile Mchiza, Whadi-ah Parker, Demetre Labadarios, Lumbwe Chola, Charles Hongoro, Khangelani Zuma, Alana T. Brennan, Peter C. Rockers, Sydney Rosen

**Affiliations:** 1 Department of Global Health, Boston University School of Public Health, Boston, Massachusetts, United Stated of America; 2 Population Health, Health Systems and Innovation, Human Sciences Research Council, Cape Town, South Africa; 3 Health Economics and Epidemiology Research Office, Department of Internal Medicine, School of Clinical Medicine, Faculty of Health Sciences, University of Witwatersrand, Johannesburg, South Africa; Loyola University Chicago, UNITED STATES

## Abstract

South Africa faces an epidemic of chronic non-communicable diseases (NCDs), yet national surveillance is limited due to the lack of recent data. We used data from the first comprehensive national survey on NCDs—the South African National Health and Nutrition Examination Survey (SANHANES-1 (2011–2012))—to evaluate the prevalence of and health system response to diabetes through a diabetes care cascade. We defined diabetes as a Hemoglobin A1c equal to or above 6.5% or currently on treatment for diabetes. We constructed a diabetes care cascade by categorizing the population with diabetes into those who were unscreened, screened but undiagnosed, diagnosed but untreated, treated but uncontrolled, and treated and controlled. We then used multivariable logistic regression models to explore factors associated with diagnosed and undiagnosed diabetes. The age-standardized prevalence of diabetes in South Africans aged 15+ was 10.1%. Prevalence rates were higher among the non-white population and among women. Among individuals with diabetes, a total of 45.4% were unscreened, 14.7% were screened but undiagnosed, 2.3% were diagnosed but untreated, 18.1% were treated but uncontrolled, and 19.4% were treated and controlled, suggesting that 80.6% of the diabetic population had unmet need for care. The diabetes care cascade revealed significant losses from lack of screening, between screening and diagnosis, and between treatment and control. These results point to significant unmet need for diabetes care in South Africa. Additionally, this analysis provides a benchmark for evaluating efforts to manage the rising burden of diabetes in South Africa.

## Introduction

Diabetes is an important cause of global morbidity and mortality.[1] The global prevalence of diabetes, 90–95% of which is type 2 diabetes,[2] is rising rapidly in many regions of the world as a result of population aging, urbanization, and changing lifestyles and dietary patterns.[3] These trends are particularly acute in low- and middle-income countries, where diabetes is projected to grow most rapidly over the next several decades.[4] Compared to other world regions, Africa currently has the lowest prevalence of diabetes, but it has the highest mortality rate due to diabetes and the highest percentage of undiagnosed cases.[5]

In South Africa, diabetes currently ranks second among the top ten leading natural causes of death, accounting for 5.4% of deaths.[6] With an adult population (aged 15+) of close to 37 million,[7] South Africa has an estimated 2.6 million people with diagnosed diabetes and a further 1.2 million people estimated to be living with undiagnosed diabetes.[8] The prevalence of diabetes in South Africa appears to be increasing over time; one recent study of urban, black South Africans found that prevalence increased from 8.0% in 1990 to 12.2% in 2008–2009.[9]

The actual prevalence of diabetes in South Africa, as well as the magnitude of unmet need for diabetes care, remains unclear, as prior estimates for South Africa are largely based on self-reported data, which do not capture undiagnosed cases and often come from sub-national samples and/or pooled estimates drawing on data from multiple countries.[10] There are currently no robust national health surveillance data to confirm self-reports, identify disparities among population groups, or generate a clear picture of the need for additional diagnosis and care.

In 2013, South Africa outlined its strategy for the prevention and control of non-communicable diseases (NCDs).[11] The strategy called for a 30% increase in the percentage of patients with controlled diabetes by 2020 but indicated that the baseline to which this increase would apply had not yet been established. Effective implementation, monitoring, and evaluation of the strategy will require high-quality nationally representative data on current NCD prevalence and uptake and outcomes of care—information that has not been previously available.

Monitoring the population level management of diabetes can be achieved using a care cascade—a method of representing the proportion of people that reach each stage of care from screening for the disease to control.[12] This type of analysis is useful in identifying the points in the cascade at which most patients are lost in order to inform policy and guide the development of future interventions.[13] Although this technique is most commonly used to analyze the HIV care continuum,[14–16] the cascade of care has recently been applied to diabetes care in the United States to visualize gaps in awareness, diagnosis, engagement, and treatment.[17] To the best of our knowledge, there are no previous applications of this approach for diabetes in the South African context.

In this study, we use data from a unique national survey of the adult population of South Africa conducted in 2011–2012 [18] to assess prevalence of diabetes and unmet need for care through a care cascade. Using this approach, we estimate gaps in screening, diagnosis, treatment, and control of diabetes for the population as a whole and for major population subgroups. We thus begin to fill the gaps in the evidence base that will allow a targeted response to the growing epidemic of diabetes in South Africa.

## Methods

This study draws on data from South African adults who participated in the first South African National Health and Nutrition Examination Survey (SANHANES-1). The SANHANES-1 is a national survey of the non-institutionalized population of South Africa conducted by the Human Sciences Research Council in 2011–2012 to measure the nutrition and health status of the population.[18,19] The survey employed a multi-stage disproportionate stratified cluster sampling design. A total of 1,000 census enumeration areas (EAs) from the 2001 population census were selected from a database of 86,000 EAs. These were stratified by province and locality type, and, in the formal urban areas, race was used as an additional stratification variable. A total of 500 EAs representative of the socio-demographic profile of South Africa were identified, and a random sample of 20 households was randomly selected from each EA, yielding an overall sample of 10,000 households. All individuals residing in selected households were eligible to participate.

The survey included an interview, a medical examination, and blood sampling for biomarker analysis. At the household level, 8,166 of the 10,000 households were occupied and contactable. These households yielded 27,580 individuals of all ages who were eligible to be interviewed and agreed to participate, 25,532 (92.6%) of whom completed the interview. Of the latter number, 12,025 (43.6%) and 8,078 (29.3%) individuals volunteered to undergo a medical examination and provide a blood sample for biomarker analysis, respectively. Additional details of SANHANES-1 methodology, content, and laboratory procedures are reported elsewhere.[19]

The analysis reported here was restricted to South Africans aged 15 and above with non-missing information on race, sex, and province. Among those who consented to providing a blood sample for biomarker analysis, 17% had missing data on HbA1c and were excluded. Additional exclusion criteria were applied in order to conduct the diabetes care cascade and analysis. Participants were excluded because of missing data on self-reports of diabetes screening and diagnosis. Of the respondents who reported a prior diagnosis, only those who reported whether they were currently taking tablets or insulin to lower their blood sugar were retained. S1 Fig depicts the full exclusion criteria resulting in an analytic sample size of 4,083.

The WHO and American Diabetes Association recommended threshold of 6.5% for HbA1c was used for the diagnosis of diabetes in the current analysis.[2] Individuals were considered to have diabetes if they had HbA1c greater than or equal to 6.5% or were currently taking either oral glycemic medication or insulin. Individuals were considered to be prediabetic if they had HbA1c between 5.7% and 6.5% and were not currently taking medication for diabetes. The American Diabetes Association recommended HbA1c target of under 7.0% was used to assess control among those with diabetes.[20]

**Fig 1:**
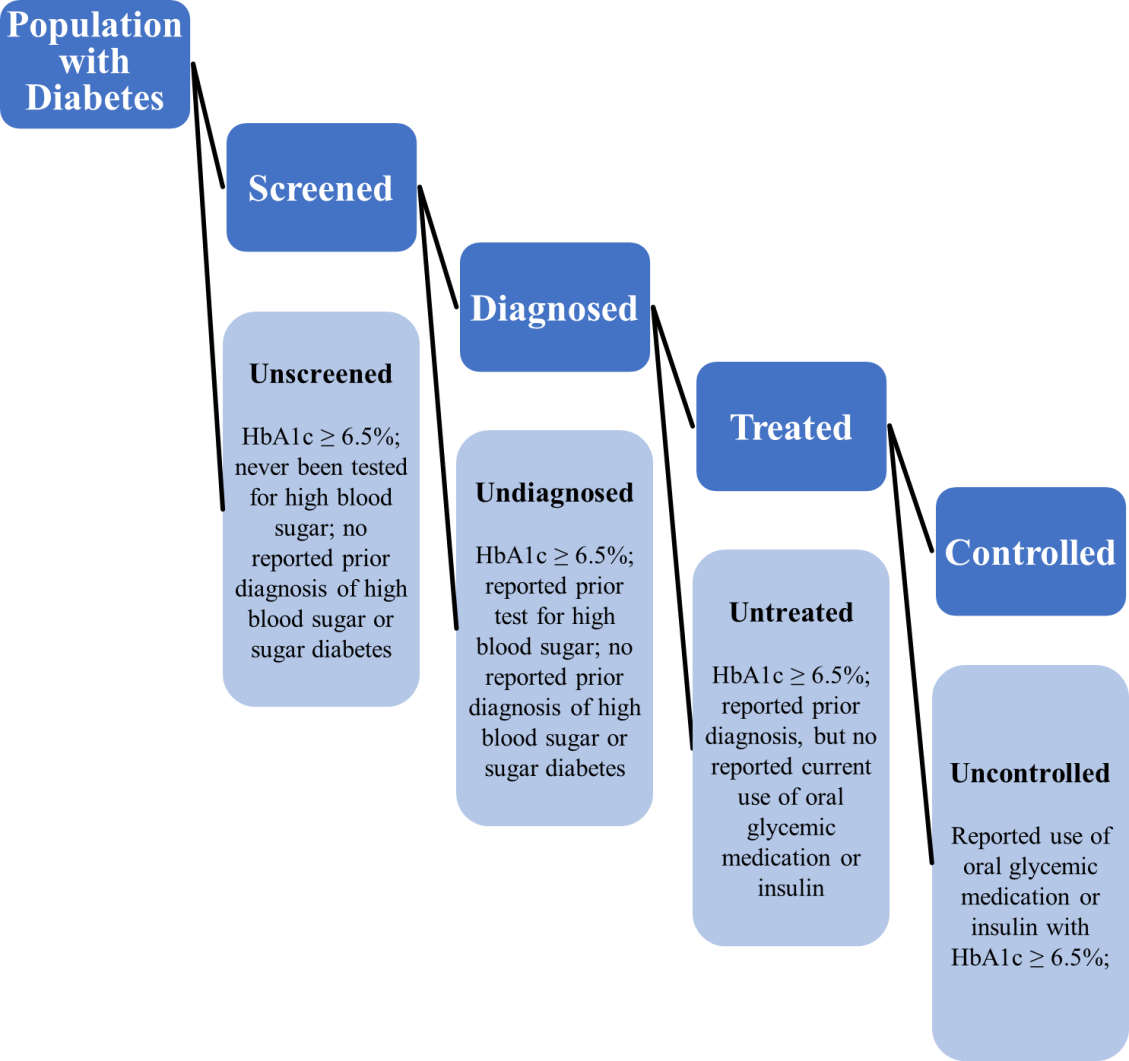
Diabetes care cascade criteria.

We developed a care cascade to examine unmet need for diabetes care and identify opportunities for intervention across the domains of screening, diagnosis, treatment, and control. This analysis entailed grouping the diabetes subpopulation into five mutually exclusive and exhaustive categories: 1) unscreened (HbA1c ≥ 6.5%; never tested for high blood sugar or sugar diabetes; no reported prior diagnosis) 2) screened, undiagnosed (HbA1c ≥ 6.5%; reported being tested ever; no reported prior diagnosis of diabetes); 3) diagnosed, untreated (prior reported diagnosis of diabetes, but no reported current use of oral glycemic medication or insulin therapy); 4) treated, uncontrolled (reported current use of oral glycemic medication or insulin therapy with HbA1c greater than or equal to 7.0%); and 5) treated, controlled (reported current use of diabetes medication with HbA1c value of less than 7.0%). The criteria for each category are summarized in Fig 1.

The five categories defined above form a diabetes care cascade that helps pinpoint where people with diabetes are lost across the continuum of care. We examined the proportion of respondents who reached each stage using the number of respondents in the subsequent stage as the denominator. For example, among those with diabetes who reported prior testing for high blood sugar, we calculated the proportion who were then diagnosed. In addition to the stages in the cascade, we defined “unmet need” as the sum of the first four of the diabetes subcategories (unscreened; screened but undiagnosed; diagnosed but untreated; treated but uncontrolled). Respondents with controlled diabetes were not considered to have unmet need for care.

To examine differences in unmet need for diabetes care across different sub-groups of the South African population, we integrated data on a number of covariates. Age, gender, race, and geographic location were ascertained by interview. Race was classified as African, Coloured, White, Indian/Asian, or other per South African standards, and the Indian/Asian and “other” categories were collapsed for analysis. Geographic status was defined using the categories urban informal, urban formal, rural informal (tribal areas), and rural formal (farms).[19] Weight and height for calculating body mass index (BMI, measured in kg/m^2^) were measured during the medical examination using standardized techniques.[19]

We then used multivariable logistic regression analysis to investigate social, demographic, and anthropometric predictors of 1) prevalent diabetes and 2) undiagnosed diabetes. Analyses for this study were performed using Stata Version 14 (StataCorp, Texas, USA). Descriptive statistics were generated using means for continuous and proportions for categorical variables. Estimates of prevalence and unmet need for diabetes care were age-standardized to the age-distribution of the South African adult population, using mid-year population estimates for 2012.[21] Age-standardization was carried out using five-year age-categories between 15 and 74 and an open-ended category of 75 and above. We used sample weights to adjust for unequal probabilities of selection and nonresponse and estimated variances using Taylor series linearization with the SVY routine. This study was based on de-identified data only. Ethics approval for this study was obtained from the Research Ethics Committee of the Human Sciences Research Council (HSRC) of South Africa and the Institutional Review Board of Boston University.

## Results

The final analytic sample included 4,083 total respondents, of whom 521 had diabetes. Descriptive statistics for the sample and the South African adult population as a whole are shown in Table 1. The sample was majority female (52.6%) and African (72.1%). Compared to the population, the analytic sample contained a smaller proportion of individuals of the African race.

**Table 1:** Characteristics of the final analytic study sample and the South African adult population aged 15 and above, 2011–2012.

	Final Analytic SANHANES Sample, 2011–2012		Mid-Year Population Estimates, 2012 Census
	No.	%	%
Sex			
Men	1459	47.4	48.1
Women	2624	52.6	51.9
Age Categories			
15–34	1791	49.1	52.0
35–54	1270	33.4	32.3
55–74	864	15.2	13.6
≥ 75	158	2.3	2.2
Race			
African	2659	72.1	77.7
White	95	13.2	10.3
Coloured	1132	11.5	9.3
Indian/Asian/Other	197	3.3	2.8
Province			
Western Cape	872	16.0	11.8
Eastern Cape	677	13.4	12.0
Northern Cape	306	2.7	2.2
Free State	347	6.9	5.4
KwaZulu-Natal	423	13.3	18.8
North West	581	7.9	6.7
Gauteng	444	28.7	25.7
Mpumalanga	271	4.3	7.5
Limpopo	162	6.7	10.0
Sample Size (n)	4083		

Sample weights were incorporated to adjust the percentage estimates in the SANHANES sample for unequal probabilities of selection and nonresponse in the laboratory component of the survey. Mid-year population estimates for 2012 were obtained from South African census data (Statistics South Africa, 2012).

The age-standardized prevalence of normal blood sugar and prediabetic blood sugar in the sample was 60.2% and 29.7% respectively. The age-standardized prevalence of diabetes (HbA1c ≥ 6.5% or currently taking medication for high blood sugar) in the sample was 10.1%. Estimates of clinical blood sugar categories by population group can be found in Table 2.

**Table 2:** Diabetes classification among South African adults aged 15 and above, 2011–2012.

	Normal HbA1c < 5.7%		Prediabetes 5.7% ≤ HbA1c < 6.5%		Diabetes HbA1c ≥ 6.5% or taking medication	
	Prev	SE	Prev	SE	Prev	SE
Ages ≥ 15						
Crude	59.0	1.5	30.3	1.4	10.7	1.1
Age-standardized	60.2	1.6	29.7	1.3	10.1	1.1
Age Categories						
15–34	74.1	1.9	20.9	1.4	5.0	1.6
35–54	50.0	2.6	39.2	2.6	10.8	1.5
55–74	36.4	3.9	39.4	3.5	24.1	2.8
≥ 75	25.6	5.2	41.5	7.8	32.9	5.1
Sex						
Men	61.2	2.4	29.1	2.1	9.7	1.7
Women	59.1	1.8	30.1	1.7	10.7	1.0
Sex by Age						
Men						
15–34	71.3	3.3	22.0	2.6	6.7	3.0
35–54	55.2	3.7	36.7	4.2	8.0	2.1
55–74	40.9	6.1	37.3	5.3	21.9	3.6
≥ 75	35.0	7.8	33.9	12.6	31.1	7.7
Women						
15–34	76.9	1.9	19.4	1.6	3.7	1.1
35–54	45.6	3.7	41.3	3.6	13.1	1.8
55–74	30.3	3.9	41.6	4.5	28.1	3.1
≥ 75	17.1	4.4	48.4	7.0	34.5	7.0
Race						
African	59.4	1.7	31.0	1.4	9.7	1.2
White	74.7	3.3	18.3	3.8	7.1	2.2
Coloured	52.4	2.4	36.3	2.4	11.3	1.4
Indian/Asian/Other	56.5	5.0	17.9	2.7	25.6	4.8
Residential Location						
Urban Formal	60.4	2.4	28.7	2.0	10.9	1.4
Urban Informal	64.2	2.8	27.2	2.3	8.6	1.6
Rural Informal	58.6	3.0	31.8	2.5	9.6	2.4
Rural Formal	59.8	3.6	32.6	2.7	7.5	2.0
BMI Category						
Underweight	61.7	4.0	37.2	4.0	1.2	0.5
Normal	67.8	2.2	26.7	1.7	5.5	1.5
Overweight	65.6	2.4	25.1	2.2	9.3	1.4
Obese	48.8	3.1	33.5	2.4	17.7	2.3

Prev = prevalence, SE = Standard Error. Normal = HbA1c < 5.7%; Prediabetes = 5.7% ≤ HbA1c < 6.5%; Diabetes = HbA1c ≥ 6.5% or currently taking medication. The following BMI categories were used: underweight (BMI < 18.5 kg/m^2), normal (18.5 ≤ BMI < 25), overweight (25 ≤ BMI < 30), and obese (BMI ≥ 30). Estimates for the overall population and by sex, race, geography and BMI were age-standardized using five-year age-categories between 15 and 74 and an open-ended category of 75 and above. Standard values were obtained from mid-year population estimates for 2012 (Statistics South Africa, 2012).

**Fig 2:**
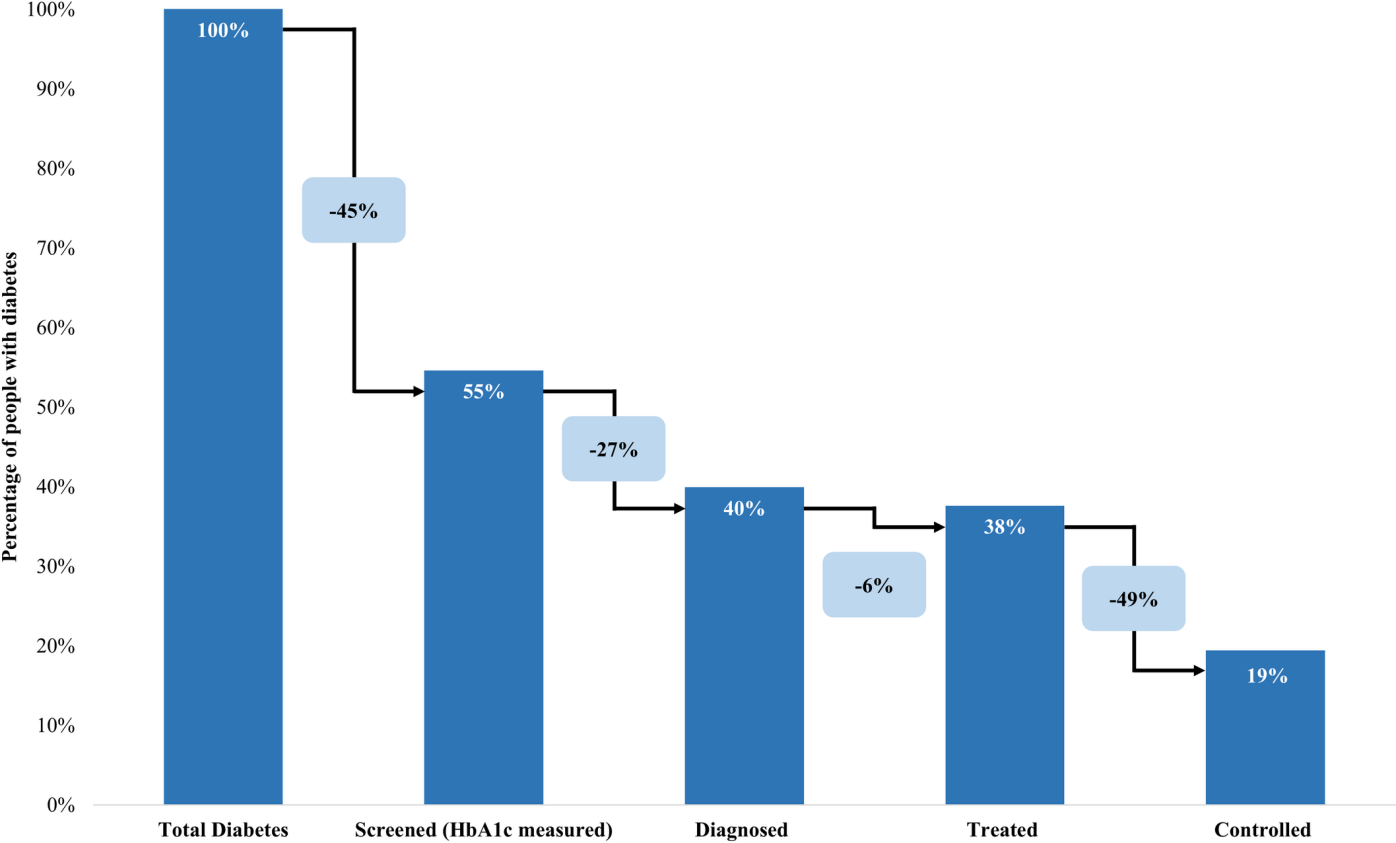
The diabetes care cascade, South Africa 2011–2012. Of those with diabetes, 55% have ever been screened for diabetes, a 45% loss. Of those who have ever had their blood sugar measured, 73% received a diagnosis of high blood sugar or sugar diabetes, a 27% loss. Of those who received a diagnosis, 94% were being treated with oral glycemic medication or insulin, a 6% loss. Of those who were currently taking medication, 51% had controlled blood sugar (HbA1c < 7.0%), a 49% loss.

Results of the diabetes care cascade are displayed in Table 3 and Fig 2. We present the distribution of diabetic respondents across the five subcategories (unscreened; screened but undiagnosed; diagnosed but untreated; treated but uncontrolled; and controlled) before discussing the transitions between stages of the care continuum.

**Table 3:** Diabetes prevalence, screening, diagnosis, treatment, and control, South African adults aged 15 and above, 2011–2012.

	Total Diabetes		Unscreened, Undiagnosed			Screened, Undiagnosed			Diagnosed, Untreated			Treated, Uncontrolled			Treated, Controlled		
	Prev	SE	Prev	SE	% of diab	Prev	SE	% of diab	Prev	SE	% of diab	Prev	SE	% of diab	Prev	SE	% of diab
Ages ≥ 15																	
Crude	10.7	1.1	3.5	0.7	32.8	2.2	0.8	20.4	0.4	0.2	4.2	2.8	0.3	26.2	1.8	0.3	16.5
Age-standardized	10.1	1.1	3.3	0.6	45.4	2.0	0.7	14.7	0.4	0.2	2.3	2.6	0.3	18.1	1.7	0.3	19.4
Age Categories																	
15–34	5.0	1.6	2.2	1.0	55.8	1.7	1.2	15.8	0.0	0.0	0.2	0.4	0.2	8.8	0.6	0.3	19.3
35–54	10.8	1.5	3.7	0.7	38.4	1.3	0.3	11.5	0.7	0.4	4.5	3.3	0.8	25.0	1.9	0.7	20.5
55–74	24.1	0.0	6.6	0.0	27.5	4.0	0.0	16.1	1.1	0.0	5.1	8.7	0.0	37.5	3.7	0.0	13.8
≥ 75	32.9	5.1	3.7	1.3	11.3	9.4	3.6	28.5	0.2	0.2	0.7	5.9	2.2	18.0	13.6	5.5	41.5
Sex																	
Men	9.7	1.7	3.3	1.2	44.0	2.5	1.4	14.8	0.4	0.3	2.7	2.2	0.5	30.1	1.3	0.4	8.3
Women	10.7	1.0	3.5	0.5	46.3	1.7	0.4	14.0	0.4	0.1	1.8	2.9	0.4	15.1	2.2	0.6	22.8
Sex by Age																	
Men																	
15–34	6.7	3.0	2.8	2.1	54.9	2.8	2.6	13.6	0.0	0.0	0.0	0.7	0.5	26.3	0.3	0.2	5.2
35–54	8.0	2.1	2.4	1.0	34.7	0.9	0.4	14.8	0.9	0.7	6.7	3.0	1.1	35.9	0.8	0.5	7.9
55–74	21.9	3.6	7.3	2.9	30.2	3.9	1.3	18.2	0.9	0.9	4.0	5.9	1.8	34.4	3.8	1.9	13.1
≥ 75	31.1	7.7	1.9	1.9	6.1	7.5	4.3	24.0	0.5	0.5	1.6	2.6	2.3	8.4	18.6	9.5	59.9
Women																	
15–34	3.7	1.1	1.7	0.6	56.5	0.8	0.6	15.0	0.1	0.1	0.9	0.2	0.1	3.8	0.9	0.6	23.7
35–54	13.1	1.8	4.8	0.9	40.8	1.6	0.4	11.0	0.4	0.3	2.3	3.3	0.8	20.6	3.1	1.4	25.3
55–74	28.1	3.1	7.2	1.7	25.0	3.9	1.1	14.1	1.5	0.7	4.5	11.6	2.1	43.6	3.9	1.1	12.8
≥ 75	34.5	7.0	5.3	2.0	15.5	11.1	5.3	32.1	0.0	0.0	0.0	8.9	3.3	25.9	9.1	3.8	26.5
Race																	
African	9.7	1.2	3.7	0.7	54.1	2.0	0.8	12.6	0.3	0.1	1.9	2.4	0.4	15.3	1.3	0.3	16.1
White	7.1	2.2	1.3	1.0	7.6	0.1	0.1	3.2	0.2	0.2	4.6	2.2	1.1	46.0	3.1	1.6	38.6
Coloured	11.3	1.4	2.8	0.6	38.4	2.4	0.6	15.5	0.9	0.7	3.8	3.3	0.7	26.8	1.8	0.7	15.6
Indian/Asian/Oth	25.6	4.8	4.9	1.6	14.4	10.5	3.8	43.5	0.3	0.2	0.9	7.0	1.6	20.2	3.0	1.2	20.9
Residential Location																	
Urban Formal	10.9	1.4	3.5	1.0	40.4	1.7	0.4	11.1	0.5	0.2	2.5	3.1	0.5	23.4	2.1	0.5	22.6
Urban Informal	8.6	1.6	4.0	1.1	68.1	0.9	0.4	5.3	0.1	0.1	5.1	1.4	0.6	8.5	2.2	1.0	13.0
Rural Informal	9.6	2.4	3.1	0.8	65.1	3.5	2.2	17.2	0.2	0.2	1.6	1.9	0.4	10.2	0.8	0.3	5.8
Rural Formal	7.5	2.0	2.6	1.2	43.2	1.1	0.5	11.1	0.1	0.1	0.5	1.5	0.7	17.9	2.2	1.0	27.3
BMI Category																	
Underweight	1.2	0.5	0.1	0.1	27.4	0.0	0.0	0.0	0.0	0.0	0.0	0.3	0.3	5.4	0.8	0.4	67.2
Normal	5.5	1.5	1.2	0.3	40.2	2.0	1.4	16.0	0.2	0.1	0.8	1.4	0.4	34.1	0.7	0.2	8.8
Overweight	9.3	1.4	3.2	0.8	41.8	0.8	0.2	19.6	1.0	0.7	4.0	2.7	0.6	17.7	1.7	0.6	16.9
Obese	17.7	2.3	6.2	1.8	47.7	3.6	0.8	13.7	0.5	0.2	1.9	4.1	0.6	17.1	3.4	1.2	19.6

Prev = prevalence, SE = Standard Error. Diabetes was defined as a Hemoglobin A1c equal to or above 6.5% or currently on treatment for diabetes. For the category of treated and controlled, HbA1c < 7.0% was used per South African standards. The following BMI categories were used: underweight (BMI < 18.5 kg/m^2), normal (18.5 ≤ BMI < 25), overweight (25 ≤ BMI < 30), and obese (BMI ≥ 30). Estimates for the overall population and by sex, race, geography, and BMI were age standardized using five-year age-categories between 15 and 74 and an open-ended category of 75 and above. Standard values were obtained from mid-year population estimates for 2012 (Statistics South Africa, 2012).

Among individuals with diabetes, nearly half were unscreened (45.4%). An additional 14.7% were screened but undiagnosed, 2.3% were diagnosed but untreated, and 18.1% were treated but uncontrolled. Only 19.4% of diabetic respondents were treated and controlled. Results of the diabetes care analysis by age, sex, race, residential location, and BMI are also presented in Table 3.

Although individuals in the age category 15–34 had lower prevalence of diabetes overall—estimated at 5.0% compared to 10.8% for those 35–54, 24.1% for those 55–74, and 32.9% for those over 75—younger people were also at higher risk of being unscreened: 55.8% of individuals with diabetes between the ages 15 and 34 were unscreened, compared to 11.3% in the age category 75+.

The age-standardized prevalence of diabetes was higher in women as compared with men (10.7% versus 9.7%). The likelihood of being unscreened was also slightly higher in women (46.3%) than in men (44.0%), while the likelihood of being treated but uncontrolled was higher in men than in women (30.1% of men with diabetes were treated but uncontrolled compared to 15.1% of women with diabetes). Women with diabetes were more likely to have controlled blood sugar than men (22.8% versus 8.3%).

Overall, the age-standardized prevalence of diabetes was higher in the non-white population, particularly among Indians/Asians/others. The proportion unscreened was also higher in non-whites compared to whites; 54.1% in Africans, 38.4% in the Coloured population and 14.4% among Indians/Asians/others, compared to 7.6% of white South Africans. A similar trend was seen for those with diabetes who were screened but undiagnosed (12.6% in Africans, 15.5% in the Coloured population, and 43.5% among Indians/Asians/others, compared with 3.2% among Whites). Whites were more likely to be both treated, uncontrolled and treated, controlled than non-white subgroups.

Although the prevalence of diabetes was highest in those living in urban formal areas, the proportion unscreened was lower in urban formal areas than in other settings (40.4% compared to 68.1% in urban informal areas and 65.1% in rural informal areas). With respect to BMI, prevalence increased monotonically between weight categories, progressing from 1.2% in those with a BMI of less than 18.5 kg/m^2^ to 17.7% in individuals with BMI 30 kg/m^2^ and above. The highest proportion of undiagnosed diabetes was found in the obese category, where 61.4% of individuals were either unscreened or screened but undiagnosed.

The transitions between stages of the diabetes care cascade are displayed in Fig 2, allowing us to pinpoint the stage at which diabetic respondents are lost. The first stage in the cascade is being screened for diabetes, which was ascertained through the question “have you ever been tested for high blood sugar or sugar diabetes?” Among those with diabetes, 54.6% reported that they had ever been screened. Among those who self-reported ever being screened, 72.5% reported that they had been previously been diagnosed (told they had high blood sugar or diabetes by a health professional). Among those who self-reported a prior diagnosis, 93.7% also reported that they were currently taking medication to treat/control their diabetes. Among those treated for diabetes, 51.2% had controlled blood sugar (HbA1c < 7.0%).

In the multivariable analysis of predictors of diabetes prevalence (Table 4), we found elevated odds ratios (OR) associated with higher age, Indian/Asian/other race, BMI in the overweight and obese range, and family history of diabetes, whereas an inverse association was found for white South Africans. In the analysis of predictors of undiagnosed diabetes (either screened or unscreened), restricted to individuals in the sample with diabetes, the Indian/Asian/other racial group had elevated risk of being undiagnosed as compared with the African racial group. Additionally, older age predicted higher likelihood of being undiagnosed. We also found that family history of diabetes was associated with a lower risk of being undiagnosed.

**Table 4:** Predictors of diabetes prevalence and diagnosis, South Africa 2011–2012.

	Predictors of diabetes				Predictors of being undiagnosed			
	OR	95% CI		P value	OR	95% CI		P value
Age Categories								
15–34	1.00				1.00			
35–54	1.51	0.69	3.30	0.30	0.80	0.30	2.08	0.64
55–74	3.55	1.53	8.21	0.00	1.74	0.66	4.57	0.26
≥ 75	4.79	1.61	14.21	0.00	2.69	0.78	9.34	0.12
Sex								
Men	1.00				1.00			
Women	0.58	0.29	1.17	0.13	0.43	0.17	1.06	0.07
Race								
African	1.00				1.00			
White	0.60	0.24	1.54	0.29	0.23	0.04	1.26	0.09
Coloured	1.38	0.79	2.41	0.26	1.12	0.56	2.23	0.75
Indian/Asian/Other	4.06	1.99	8.29	0.00	4.23	1.70	10.53	0.00
Residential Location								
Urban Formal	1.00				1.00			
Urban Informal	0.71	0.37	1.37	0.31	0.67	0.28	1.59	0.36
Rural Informal	1.15	0.43	3.02	0.78	1.47	0.43	5.08	0.54
Rural Formal	1.06	0.50	2.27	0.87	1.04	0.33	3.28	0.95
BMI Category								
Normal	1.00				1.00			
Overweight	2.60	1.19	5.68	0.02	2.40	0.82	7.03	0.11
Obese	5.73	2.37	13.86	0.00	7.28	2.09	25.33	0.00
Family History of Diabetes	2.33	1.46	3.72	0.00	0.83	0.48	1.44	0.50

OR = odds ratio; BMI = body mass index; CI = confidence interval. Diabetes was defined as a Hemoglobin A1c equal to or above 6.5% or currently on treatment for diabetes. The analysis of predictors of having undiagnosed diabetes was restricted to those with diabetes. “Undiagnosed” here refers to all diabetic respondents who have never been screened for high blood sugar and those who have been screened but never received a diagnosis. The following BMI categories were used: normal (18.5 ≤ BMI < 25), overweight (25 ≤ BMI < 30), and obese (BMI ≥ 30).

## Discussion

In this study, using data from a unique national survey which combined questionnaires with medical examination and biomarker analysis, we report the first nationally representative estimate of the burden of diabetes among South African adults. Our analysis revealed both high diabetes prevalence and substantial unmet need for diabetes care in the South African population, as well as notable disparities across groups. Of the 10.1% of those aged 15+ with diabetes, only 19.4% were treated and controlled; 45.4% were unscreened, 14.7% were screened but undiagnosed, 2.3% were diagnosed but untreated, and 18.1% were treated but uncontrolled.

Our estimates indicate a higher prevalence of diabetes than previously reported by the National Income Dynamics Study (NIDS) in 2012 for South Africans aged 40+ (18.9% prevalence for SANHANES compared to 14.3% for NIDS).[22] However, the NIDS relied exclusively on self-reports of diabetes whereas our analysis incorporated biomarker analysis. Our estimates are comparable to those reported by the NCD Risk Factor Collaboration, which calculated a pooled prevalence estimate for diabetes of 9.0% for men and 11.8% for women aged 18+ in 2010 using biomarker data from six community-level studies in South Africa.[1] Similarly, we report a prevalence of 10.0% for men and 10.7% for women of the same age range in 2011–2012.

Estimates presented in this analysis indicate a slightly lower diabetes prevalence but substantially greater unmet need for diabetes care in South Africa than in the United States. Recent estimates for the U.S. place age-standardized diabetes prevalence at 12.3% for adults aged 20+,[23] compared to our estimate of 11.6% for the same age range in South Africa. In the U.S., however, the proportion undiagnosed is estimated to be 27.8%,[23] far lower than our estimate of 53.7% for South Africa. These figures suggest that South Africa faces a greater burden of disability, and potentially mortality, from diabetes than does the U.S., which has far greater resources with which to identify and care for chronic disease patients.

The diabetes care cascade reveals that one of the key gaps in the national management of diabetes is proper screening; nearly half of diabetic respondents reported never even having their blood sugar measured. Of those with diabetes who reported prior screening/testing, 72.5% received a diagnosis, indicating another significant loss between the stage of screening and diagnosis. Although some of the respondents who reported having their blood sugar measured may have been screened before becoming diabetic, 79.2% of the people who were screened but undiagnosed reported that they had their blood sugar measured within the last year. In total, only 39.9% of those with diabetic level HbA1c reported awareness of their condition.

In addition to the poor rates of screening and diagnosis, the care cascade suggests that a gap exists in terms of effective treatment with diabetes medication. Among those who reported use of oral glycemic medication or insulin, only 51.2% had controlled blood sugar. Contributing factors to this effective treatment gap likely include lack of health education and poor medicine adherence on the part of patients.[24–26] Low levels of adherence have been documented for several chronic disease treatments in South Africa, including antiretroviral therapy (ART) and tuberculosis regimens.[27–29] Other factors which contribute to the problem of ineffective treatment include lack of diabetes expertise among healthcare providers, who fail to impart quality prevention, treatment, and management education to their patients,[30] and interruptions in the supply of medicines available in public sector clinics, which leads diabetes treatment to be postponed or forfeited.[31]

Several factors may contribute to the high rate of unmet need for diabetes care in South Africa across the care continuum, among them problems with access, health-seeking behavior, and health system quality. Insufficient access to health care services is widespread, with the most important barriers to access relating to low socio-economic status, racial background, lack of health insurance,[24] and the costs incurred in traveling to clinics, particularly among black South Africans and those living in rural areas.[25] Inadequate demand for healthcare is also an obstacle, particularly among males and young adults.[32]

Many of these barriers are highlighted in a recent qualitative study among low-income black women in Soweto, in which a large proportion of participants had no health insurance and sought diabetes care in public health facilities with limited availability of diabetes counseling and treatment and low quality of care.[33] Similarly, Isaacs et al. found that the quality of care for diabetes and hypertension in primary health care facilities in the Cape Town Metropole was poor compared to the national guideline recommendations.[34] The inadequacy of national NCD surveillance further hampers the health system’s ability to identify and respond to unmet healthcare needs.

Strengths of the current study included use of data from a large national sample and the measurement of Hemoglobin A1c, an objective criterion for defining diabetes, which allowed us to obtain estimates of the total prevalence of diabetes in South Africa as well as to investigate control status. Another strength is the use of a care cascade to identify gaps in the population-level management of diabetes.

A limitation of this study was the low response rate to the laboratory component of the SANHANES, since testing was conducted in referral sites, not at the point of survey administration (in contrast, for example, to the US NHANES, which employed mobile examination units). We conducted a bias analysis to compare demographic characteristics of the final analytic sample and those excluded between interview and analysis (S1 Table). This table showed that those of the African race and aged 15–34 were disproportionately excluded between interview and analysis, a possible source of selection bias during the laboratory stage. Furthermore, if those who are symptomatic and/or lack routine access to care were more likely to go to the laboratory appointment, we may have overestimated prevalence and unmet need. However, our estimates of diabetes prevalence are close to prior estimates suggesting this is not a major source of bias in this analysis. Nevertheless, the findings of the present study should be confirmed in future national studies. The sensitivity of estimates to alternative criteria for defining diabetes, such as fasting plasma glucose, should also be explored.

Until recently, health policy and programming in South Africa have largely focused on infectious and communicable diseases, and the majority of all health expenditure has been directed to the prevention and management of these diseases. With the recent launch of the national NCD strategy, however, momentum to tackle the burden of NCDs is growing. Given South Africa’s large past investments in chronic HIV care, the national NCD strategy outlines an approach that integrates diabetes care into these existing systems.

This study documents high levels of unmet need for diabetes care among South African adults with diabetes and points to stages in the diabetes care continuum with the biggest gaps in population-level management. The current estimates should serve as a benchmark for evaluating the effectiveness of the proposed reforms, particularly the re-engineering of primary care, and motivate policies aimed at redressing unmet need for diabetes care in South Africa.

## Supporting information

S1 Fig Exclusion criteria for diabetes care cascade anaylsis. (TIF)

S1 Table Characteristics of the analytic study sample and the excluded participants. The columns "All Excluded Observations from SANHANES interview sample" show all people excluded between the adult interview sample and the final analytic sample including those who did not complete the lab portion of the exam. The columns "Excluded observations from the SANHANES lab sample" show only the observations that were excluded from the lab sample based on criteria unique to this analysis. Sample weights were incorporated to adjust the percentage estimates in the SANHANES samples for unequal probabilities of selection and nonresponse in the laboratory component of the survey.
(PDF)
