# Supplementary figures and images for: Prevalence and unmet need for diabetes care across the care continuum in a national sample of South African adults: Evidence from the SANHANES-1, 2011-2012
Source: PLoS One. 2017 Oct 2;12(10):e0184264. doi: 10.1371/journal.pone.0184264 (PMC5624573; doi:10.1371/journal.pone.0184264)

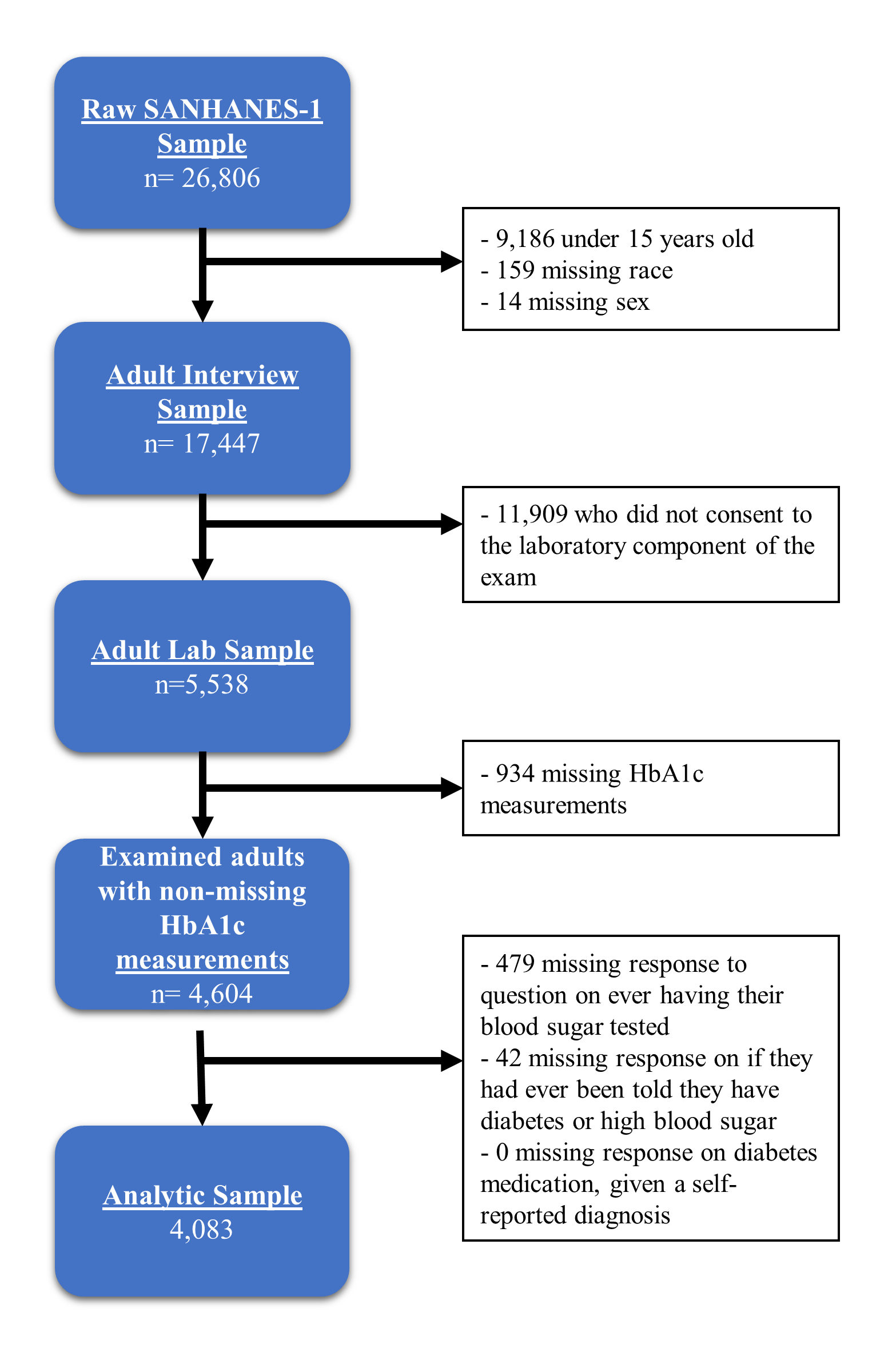

Supplement: S1 Fig — (TIF) [file pone.0184264.s001.TIF]
