# Supplementary material for: Prevalence and unmet need for diabetes care across the care continuum in a national sample of South African adults: Evidence from the SANHANES-1, 2011-2012
Source: PLoS One. 2017 Oct 2;12(10):e0184264. doi: 10.1371/journal.pone.0184264 (PMC5624573; doi:10.1371/journal.pone.0184264)
Supplement: S1 Table — The columns "All Excluded Observations from SANHANES interview sample" show all people excluded between the adult interview sample and the final analytic sample including those who did not complete the lab portion of the exam. The columns "Excluded observations from the SANHANES lab sample" show only the observations that were excluded from the lab sample based on criteria unique to this analysis. Sample weights were incorporated to adjust the percentage estimates in the SANHANES samples for unequal probabilities of selection and nonresponse in the laboratory component of the survey. (PDF) [file pone.0184264.s002.pdf]

S1 Table. Characteristics of the analytic study sample and the excluded participants

	All Excluded Observations from SANHANES Interview Sample, 2011-2012		Excluded Observations from SANHANES Lab Sample, 2011-2012		Final Analytic Sample SANHANES Lab Sample, 2011-2012	
	**No.**	**%**	**No.**	**%**	**No.**	**%**
Sex						
Men	6037	48.0	491	37.2	1459	47.4
Women	7327	52.0	964	62.8	2624	52.6
Age Categories						
15-34	6683	53.2	675	58.0	1791	49.1
35-54	4170	32.3	428	29.8	1270	33.4
55-74	2084	11.9	286	10.3	864	15.2
≥ 75	427	2.6	66	1.9	158	2.3
Race						
African	8825	77.2	1163	86.5	2659	72.1
White	701	11.0	20	5.2	95	13.2
Coloured	2413	8.3	198	4.8	1132	11.5
Indian or Asian	1425	3.5	74	3.5	197	3.3
Province						
Western Cape	1670	10.6	107	4.2	872	16.0
Eastern Cape	1226	10.1	183	7.9	677	13.4
Northern Cape	815	2.1	64	1.2	306	2.7
Free State	643	4.0	106	2.4	347	6.9
KwaZulu-Natal	2610	20.8	329	31.2	423	13.3
North West	1495	6.5	88	2.4	581	7.9
Gauteng	2494	26.3	138	23.5	444	28.7
Mpumalanga	1229	8.1	240	11.2	271	4.3
Limpopo	1182	11.4	200	16.0	162	6.7
Sample Size (n)	13364		1455		4083	

The columns "All Excluded Observations from SANHANES interview sample" show all people excluded between the adult interview sample and the final analytic sample including those who did not consent to the lab portion of the exam. The columns "Excluded observations from the SANHANES lab sample" show only the observations that were excluded from the lab sample based on criteria unique to this analysis. Sample weights were incorporated to adjust the percentage estimates in the SANHANES samples for unequal probabilities of selection and nonresponse in the laboratory component of the survey.
